# The value of diffusion tensor imaging for differentiating autism spectrum disorder with language delay from developmental language disorder among toddlers: Erratum

**DOI:** 10.1097/MD.0000000000023796

**Published:** 2020-12-18

In the article, “The value of diffusion tensor imaging for differentiating autism spectrum disorder with language delay from developmental language disorder among toddlers”,^[1]^ which appears in Volume 98, Issue 14 of *Medicine*, Figure 1 and references to it have been removed as the authors did not obtain permission to use the figure from the authors of “Arcuate Fasciculus in Autism Spectrum Disorder Toddlers with Language Regression.” The change does not affect the conclusions or data in the paper.
